# Humorous cognitive reappraisal: More benign humour and less "dark" humour is affiliated with more adaptive cognitive reappraisal strategies

**DOI:** 10.1371/journal.pone.0211618

**Published:** 2019-01-31

**Authors:** Corinna M. Perchtold, Elisabeth M. Weiss, Christian Rominger, Kurt Feyaerts, Willibald Ruch, Andreas Fink, Ilona Papousek

**Affiliations:** 1 Department of Psychology, University of Graz, Graz, Austria; 2 Department of Linguistics, University of Leuven, Leuven, Belgium; 3 Department of Psychology, University of Zurich, Zurich, Switzerland; Western Sydney University, AUSTRALIA

## Abstract

The capacity to find humorous perspectives in aversive situations may outline a helpful strategy in the context of cognitive reappraisal. Yet, research suggested that some people produce more adaptive humour than others. At the same time, not all forms of cognitive reinterpretation seem to be unequivocally beneficial. The present study aimed to investigate specific cognitive reappraisal strategies that individuals employ in humorous reappraisal of adverse events. In a sample of 95 participants, the use of cognitive reappraisal sub-strategies was assessed in a behavioural test in which participants were required to generate a series of humorous reappraisals of self-relevant, threatening events. These reappraisal sub-strategies (three positive reinterpretation strategies, three de-emphasising strategies) were then related to the habitual use of different kinds of humour as well as the broader DSM-5 personality trait domains and well-being in terms of depressive experiences, assessed by self-report questionnaires. While no robust relationships were found for reappraisal strategies based on de-emphasising, sub-strategies within the positive reinterpretation category showed specific and contrasting associations with the examined traits. Findings indicated that the ability to produce humour is only linked to a favourable pattern of reappraisal strategies when manifested in benign forms of humour. Specific relations also emerged for the broader personality traits. The study suggests that some characteristics that advance the use of benign humour also benefit adaptive emotion regulation. The opposite seems to be true for malicious, or "dark" humour. The introduced behavioural approach to the analysis of humorous cognitive reappraisal may prove useful also in future related research.

## Introduction

It has been suggested that the production of humour may help to maintain psychological well-being by supporting more positive appraisal of stressful events [1–3]. Empirical research has shown that greater use of humour is associated with appraising events in a more positive, less threatening manner [4–6]. Along these lines, the use of benign humour has been identified as an effective means in the context of volitional cognitive reappraisal [7–9]. Cognitive reappraisal is conceptualized as the process of reinterpreting the subjective meaning of an emotionally evocative event, thereby changing its emotional impact [10], and is considered a particularly powerful coping strategy [11,12]. The effectiveness of humour in the context of cognitive reappraisal was attributed to the perspective change on a negative event, which facilitates greater emotional distance and exchanges negative for positive emotions due to absurd elements and incongruity-resolution that is inherent to humour processing [8,9]. Since humorous cognitive reappraisal, if successful, putatively outperforms serious reappraisal in the downregulation of negative emotions [9], the present study aimed for a more in-depth investigation of the nature of humour in cognitive reappraisal. More precisely, the study rationale was to determine how specific cognitive reappraisal strategies used in humorous cognitive reappraisal of threatening events relate to individuals' habitual use of benign and dark humour, including potential relationships with relevant personality traits.

In previous empirical investigations directly addressing the use of humour in cognitive reappraisal, negative pictures were provided with pre-specified humorous interpretations [7], or participants were instructed to humorously reappraise negative pictures [8,9], with the impact assessed by affect ratings. While having provided vital evidence, these approaches did not determine to which extent participants were able to produce cognitive reappraisals at all and lacked information on the specific structure of the generated reappraisals. In order to test for individuals’ generation of certain types of cognitive reappraisals when using humour, we used a modified version of the Reappraisal Inventiveness Test (RIT), a recently introduced performance test for cognitive reappraisal generation [13–16]. In the modified humour version, participants were confronted with self-relevant, threatening situations and instructed to produce as many different *humorous* cognitive reinterpretations as possible in order to downregulate their experienced stress and anxiety. The RIT offers the unique possibility of categorizing individuals’ reappraisal ideas into a number of specific predefined sub-strategies that go beyond the more general distinction in literature, which is mostly between self-focused, detached reappraisal versus situation-focused, positive reinterpretation [17,18]. While detached reappraisals typically refer to reframing a stimulus in a distanced, unemotional way and are oriented toward the perspective of an indifferent observer or third person, positive reinterpretations focus on reframing negative situational aspects in a more positive light or on seeing potential positive outcomes of critical situations [18,19]. This differentiation is important because it is debated that some types of reappraisal may be more effective than others [18,20]. Accordingly, the generation of positive reinterpretations in particular was related to better stress resilience and emotion regulation success [21–23]. Furthermore, a recent study showed that a greater use of positive reinterpretations but not detached reappraisals for anger-eliciting events was linked to less chronic stress experience [16]. Thus, a primary research question of this study was whether some reappraisal strategies, when coupled with humour, appear more favourable than others in terms of relationships with psychological well-being. The reappraisal category scheme of the RIT and the data of the present study allowed to distinguish the implementation of three sub-strategies within the positive reinterpretation category (finding general positive aspects, worst-case comparisons, interpreting a disadvantage as an advantage) and three sub-strategies within the de-emphasising category (finding alternative explanations, trivializing the problem, handing over responsibility) in humorous cognitive reappraisal of threatening events. This more precise differentiation of reappraisal sub-strategies embedded in the broader categories accounts for the fact that despite sharing an underlying goal (e.g., positive reinterpretation vs. de-emphasising), sub-strategies from the same category may entail markedly different cognitive re-constructions of target situations (see also [24]). While, for instance, in the RIT the positive reinterpretation sub-strategy of finding general positive aspects emphasises positive aspects of a situation that are not directly related to the threatening experience (e.g., appreciating one's accomplishments of the day), the positive reinterpretation sub-strategy of worst-case comparisons refers to imagining an even worse scenario than the given one and as a result, reappraise the latter as less negative (for more details, see S1 Appendix). For this reason, different reappraisal sub-strategies may be associated with individual traits and well-being in different ways.

A more differentiated view may also be important when it comes to the kinds of humour that people habitually employ. While there are various ways to categorize humorous behavior [25], common ground has been established in differentiating between the use of benign humour and malicious humour. Benign humour is usually aimed at brightening others up and pointing up funny sides of adversities in a good-natured manner, while malicious, or “dark” humour is based on injurious, mean-spirited goals and attitudes [26–28]. Research suggested that using good-natured humour for reappraisal yields better up-regulation of positive and down-regulation of negative emotions than mean-spirited humour [8]. However, no efforts have been made so far to ascertain whether the use of different kinds of humour drives the generation of specific types of cognitive reappraisals. This constitutes an important step in bridging research on humour, emotion regulation, and well-being, considering that different types of cognitive reappraisals may be linked to more or less favourable outcomes. In the present study, we used a psychometrically reliable self-report instrument for the assessment of the habitual use of different kinds of humour [28–30], for which the distinction between benign and malicious, or "dark" humour (encompassing sarcasm, cynicism, and irony) was neurophysiologically validated [26]. This corresponds to the research question whether the habitual use of benevolent or "darker" humour is related to the implementation of specific reappraisal strategies in humorous cognitive reappraisal.

Finally, it seems additionally important to shed some light on associations with more general personality traits. There is preliminary evidence that individuals with higher expressions of maladaptive personality features may be prone to not selecting the most effective strategies for coping with emotionally evocative events [31,32]. Previous research also pointed to associations of personality traits and personality disorders with humour production ability and the structure and content of produced humour [33–35]. Thus, in the present study, we also evaluated potential links between the implementation of certain strategies in the humorous cognitive reappraisal task and the DSM-5 personality trait domains (Personality Inventory for the DSM-5; [36]). These domains represent a maladaptive extension of the classic five-factor model of personality [37,38]. Both the DSM-5 personality trait domains and the classic Big Five traits (encompassing openness to experience, conscientiousness, extraversion, agreeableness, and neuroticism) were associated with preferences for certain kinds of humour in previous research [39–41]. More specifically, the DSM-5 personality domains of negative affectivity and detachment seem to be negatively associated with habitual use of benign humour, whereas disinhibition is positively associated with habitual use of injurious humour [41]. Yet, these maladaptive extensions of personality traits have not been linked to humorous cognitive reappraisal to date. This motivated our research question whether the implementation of specific reappraisal strategies in humorous cognitive reappraisal may be related to potentially relevant personality traits.

Taken together, the aim of this study was to explore potential links between the habitual use of benign and malicious humour as well as the broader DSM-V personality trait domains and the behaviourally assessed implementation of specific strategies in the generation of humorous cognitive reappraisals of threatening events. To get apotential indication of their adaptive value in real life, it was also examined whether the relative use of any of these reappraisal strategies in humorous cognitive reappraisal may be linked to psychological well-being in terms of depressive experiences. In light of the meaningful links between humour, emotion regulation, and well-being [8,9] as well as humour styles and personality traits [39–41], and the compelling question what types of humour and cognitive reappraisal strategies are actually adaptive, we saw merit in taking the exploratory approach to linking all these aspects, and suggest an integrative framework that may prove useful for future related research.

## Methods

### Participants

The sample comprised 95 participants (57 female, 38 male), aged between 17 and 34 years (*M* = 21.4, *SD* = 4.1). Participants were recruited online via social media, and offline via posters at several university and high school and college campuses. Interested individuals were phoned to check for exclusion criteria and arrange an appointment. Levels of education were: less than high school (33), high school graduate (45), university degree (17). No participant reported using drugs or psychoactive medication and none had participated in an experiment using the RIT [13] before. The study was approved by the Ethics Committee of the University of Graz, approval number GZ. 39/43/63 ex 2015/16. Participants gave their written consent to participate in the study. After receiving general instructions, participants completed the adapted Reappraisal Inventiveness Test and the questionnaires.

### Reappraisal Inventiveness Test, humorous adaptation

The original RIT [13] confronts individuals with adverse emotional situations typically occurring in their everyday lives. Participants are instructed to imagine the situation happening to them and to generate and write down as many different ways as possible to appraise the situation in a way that diminishes their negative emotions. In the present study, four vignettes depicting anxiety-eliciting situations [42] were presented one at a time on separate pages and supplemented by a picture in order to make them more vivid. For each vignette, participants were given 20 seconds to imagine the situation happening to them and then turn to the next page at the signal of the experimenter. Next, participants wrote down as many humorous ways as possible to think about the situation in a way that diminishes anxiety until the allotted time of 3 min per situation had elapsed. In the *office* item of the RIT, for instance, participants face the following situation: *“Late at night*, *you are the only one left working at the office*. *As you are sitting at your desk*, *suddenly all the lights on your floor switch off”*. On average, participants generated *M* = 11.44 (*SD* = 3.52) valid (distinguishable) reappraisals. Note that this number is lower compared to previous studies using the original RIT [13,15] which is due to the higher difficulty of producing reappraisals that are also humorous in nature [9].

In order to determine to which extent the participants actually succeeded in producing (explicable) humour in their cognitive reappraisals as objectively as possible, each valid reappraisal was rated according to the agreement of three authors (C.R., K.F., I.P.) whether it followed an identifiable humour structure (1087 reappraisals were rated in total). Reappraisals were rated as "humorous" if they could be classified as either (a) incongruity-resolution humour, comprising an unexpected incongruity which could be resolved through the punch line; (b) nonsense humour, comprising an incongruity which was left unresolved; (c) disparagement humour without typical incongruity, including sarcastic, cynical, and scoffing statements (see [43–45]). They were rated as "non-humorous" reappraisals if they (d) had no identifiable humorous structure, i.e., comprised no humour in the classical sense. Importantly, this classification focused on the linguistic structure of the reappraisals and was conducted independently from the extent to which the raters perceived them funny or the subjective funniness ratings of other raters. On average, 36.3% (*SD* = 20.5) of the valid reappraisals were found to have an identifiable humorous structure. *M* = 12.0% (*SD* = 13.4), *M* = 4.6% (*SD* = 6.7), *M* = 19.7% (*SD* = 16.5) of the reappraisals were classified as incongruity-resolution (a), nonsense (b), and disparagement humour (c), respectively. To confirm their appreciation as humorous, the funniness of reappraisals classified as "humour" (403 reappraisals in total) was rated by eight independent raters on a scale from 0 (not funny at all) to 3 (extremely funny). The average rating was *M* = 1.26 (*SD* = 0.36), which was significantly different from zero (not funny), *t*(89) = 32.8, *p* < .001. The interrater-reliability was ICC = .73.

After completion of all vignettes, participants rated the extent of anxiety they would experience when confronted with the depicted situations (7-point scales ranging from 0 ‘not anxious at all’ to 6 ‘very anxious’). Ratings were *M* = 2.62 (*SD* = 1.66), *M* = 3.68 (*SD* = 1.74), *M* = 2.92 (*SD* = 1.56) and *M* = 2.80 (*SD* = 1.77). In one-sample t-tests, ratings for all vignettes differed significantly from zero (t-values ranging from 15.4 to 20.6, all p-values < .001), indicating that all situations were indeed perceived as anxiety evoking.

### Categorisation of reappraisal strategies

Each valid reappraisal (1087 in total) was categorised according to the standard category scheme of the RIT [13,42]. The RIT allows for the scoring of two main categories: positive reinterpretations (situation-focused reappraisal) and de-emphasising (self-focused reappraisal), both of which are composed of several sub-strategies. In this study, the total numbers of reappraisals for three sub-strategies of the positive reinterpretation category and three sub-strategies of the de-emphasising category [42] were calculated. Only the top three sub-strategies of each category that participants used most were included in the analyses. The other sub-strategies were excluded due to a lack of respective reappraisals generated by the participants. The three included sub-strategies for positive reinterpretation were: finding general positive aspects (α = .71, *M* = 0.62, *SD* = 0.81), worst-case comparisons (α = .75, *M* = 0.52, *SD* = 0.70), and interpreting a disadvantage as an advantage (α = .80, *M* = 1.26, *SD* = 1.21). The three sub-strategies for de-emphasising were: finding alternative explanations for the threat (α = .82, *M* = 3.94, *SD* = 2.68), trivializing the problem (α = .73, *M* = 0.81, *SD* = 1.27), and handing over responsibility (α = .68, *M* = 0.64, *SD* = 0.51). See S1 Appendix for details. Responses were independently rated by two experimenters. Inter-rater reliabilities were ICC = .74, ICC = .67, and ICC = .70 for the positive reinterpretation sub-strategies of finding general positive aspects, worst-case comparisons, and interpreting a disadvantage as an advantage, and ICC = .92, ICC = .76, and ICC = .75 for the de-emphasizing sub-strategies of finding alternative explanations, trivializing the problem, and handing over responsibility, respectively.

### Self-report measures

#### Habitual use of humour

In the CSM (Comic Style Markers, previously 8 Schmidt-Hidding Comic Styles or 8SHCS) [28,29], participants rate the extent to which 48 statements apply to the way they typically express humour on a 7-point Likert scale from 0 (strongly disagree) to 6 (strongly agree). It comprises eight subscales (6 items each), of which only four were used in the present study. The "benign humour" subscale was used to assess the use of good-natured humour (α = .70, *M* = 4.31, *SD* = 0.89). Additionally, a composite score capturing the use of malicious, or "dark" humour was calculated averaging across the "cynicism", "sarcasm", and "irony" subscales (α = .83, *M* = 3.32, *SD* = 1.29; α = .83, *M* = 3.03, *SD* = 1.39; α = .78, *M* = 3.92, *SD* = 1.18; see [25]).

#### DSM-5 personality trait domains

The Personality Inventory for DSM-5 (PID-5, German version; [46]) is a 220-item questionnaire assessing personality traits according to the DSM-5 trait model (Section III, Emerging Measures and Models, Criterion B; [47]). Items are rated on four-point Likert scales, from 0 (very false or often false) to 3 (very true or often true). The PID-5 consists of 25 trait facet scales, comprising five broad personality domains. Domain scores were calculated by averaging the facet scores contributing primarily to the specific domain as instructed by the American Psychiatric Association (Negative Affect, α = .79, *M* = 0.99, *SD* = 0.50: Emotional Lability, Anxiousness, Separation Insecurity; Detachment, α = .82, *M* = 0.61, *SD* = 0.49: Withdrawal, Anhedonia, Intimacy Avoidance; Antagonism, α = .83, *M* = 0.94, *SD* = 0.51: Manipulativeness, Deceitfulness, Grandiosity; Disinhibition: α = .74, *M* = 0.95, *SD* = 0.42; Irresponsibility, Impulsivity, Distractibility; Psychoticism, α = .80, *M* = 0.99, *SD* = 0.48: Unusual Beliefs and Experiences, Eccentricity, Perceptual Dysregulation). Domain and facet scores also show adequate variability and validity in non-clinical community and student samples with scores in the lower ranges of the scales [48–51].

#### Depression

The Center for Epidemiologic Studies Depression Scale (CES-D, German version; [52]) is comprised of 20 items, rated from 0 (rarely or none of the time–less than 1 day) to 4 (most or all the time– 5 to 7 days; α = .89). It refers to mood and attributions over the past week and is designed for measuring sub-clinical depressive experiences in the general population [53]. Scores ranged from 1 to 48 (*M* = 15.71, *SD* = 10.21).

### Statistical analysis

The main research questions were tested using standard multiple regression analyses. First, the six eligible reappraisal sub-strategies (three positive reinterpretation strategies, three de-emphasising strategies) were correlated with participants’ propensity for using benign or malicious humour in two analyses: In each of these two analyses, the six reappraisal strategies were used as the predictors. In one analysis, the propensity for using benign humour was used as the dependent variable while the use of malicious humour served as the dependent variable in the second. (Production of the two humour types is not mutually exclusive). The six reappraisal strategies were simultaneously entered in multiple regression analyses to determine whether individuals' habitual use of humour was related to the predominant implementation of certain reappraisal strategies (compared to others). Five analyses were used to correlate the five PID-5 personality domains with the relative use of the six reappraisal strategies in the test: In each of these analyses, the six reappraisal strategies were used as the predictors, and one of the personality domains was used as the dependent variable, respectively. Finally, it was examined with one analysis which use of a particular reappraisal sub-strategy was linked to depressive experiences (predictors: the six reappraisal strategies, dependent variable: depressive experiences). The applied multiple regression approach allowed to examine whether habitual use of humour, broader personality, and depression were related to unique variance of one specific reappraisal strategy independently from that of the other strategies, that is, to the relative greater use of one specific strategy compared to use of the other strategies. That is, the semi-partial correlation (*sr*) gained in the multiple regression analyses informs about the relationship between the dependent variable in the model and the predominant implementation of a certain reappraisal strategy, compared to others. The zero-order correlation (*r*), by contrast, informs about the relations of habitual use of humour, broader personality, and depression to the absolute productivity regarding a reappraisal strategy (including overlapping variance among the strategies, i.e., not relative to the individual's productivity regarding any of the other strategies or the individual's general productivity in generating humorous reappraisals). The semi-partial correlations are more relevant in the present context. Yet, we report both the semi-partial as well as the zero-order correlations, to provide the full information. As the rarely chosen categories did not enter the analyses, we do not interpret the multiple correlation coefficient R and its significance (F-test) as correlation with the individual's overall capability to generate (humorous) cognitive reappraisals in the sense of the original Reappraisal Inventiveness Test.

Supplementary analyses included intercorrelations between the habitual use of benign and malicious humour and the DSM-5 personality trait domains; as well as correlations with the rate of reappraisals classified as "humorous", serving as a proxy of the general ability to produce humour in the context of cognitive reappraisal. Results were considered statistically significant, if *p* < .05 (two-tailed). However, exact p-values are given for all analyses to make them fully open to scrutiny.

## Results

### Reappraisal strategies implemented in the generation of humorous reappraisals of threatening events and habitual use of humour

Individuals with greater self-reported use of benign humour (*sr* = .21, *p* = .049) and individuals with lower habitual use of malicious humour (*sr* = -.28, *p* = .007) produced a greater share of one specific type of the "positive reinterpretation" category, that is, reappraisals featuring general positive aspects in the situation. The unchanged size of the semi-partial correlations compared to the zero-order correlations (*r* = -.24, *r* = .21) indicates that overlapping variance among the strategies, that is, the more general productivity in generating cognitive reappraisals of any type did not play a role in this relationship. No significant associations of the habitual tendency to use mean-spirited or good-natured humour to the implementation of any of the other reappraisal strategies emerged (Table 1). The correlation between the habitual use of benign and mean-spirited humour was *r* = .30 (*p* = .013).

**Table 1:** Correlations between reappraisal strategies implemented in the generation of humorous reappraisals of threatening events and habitual use of benign and malicious humour.

		Malicious humour			Benign humour		
Reappraisal strategies		*r (p)*	*sr (p)*	*B (SE)*	*r (p)*	*sr (p)*	*B (SE)*
**Positive re-interpretation**	General positive aspects	**-.24 (.019)**	**-.28 (.007)**	-.38 (.14)	**.21 (.040)**	**.21 (.049)**	.25 (.12)
	Worst-case comparison	.05 (.608)	.15 (.128)	.24 (.16)	.05 (.607)	-.02 (.844)	-.03 (.14)
	Disadvantage as advantage	.06 (.587)	.08 (.442)	.07 (.09)	.02 (.842)	.02 (.886)	.01 (.08)
**De-emphasising**	Alternative explanation	-.14 (.175)	-.17 (.086)	-.07 (.04)	-.04 (.700)	-.04 (.732)	-.01 (.04)
	Trivialising the problem	-.05 (.610)	-.07 (.506)	-.06 (.08)	.02 (.862)	.05 (.620)	.04 (.08)
	Handing over responsibility	.12 (.230)	.15 (.146)	.45 (.30)	-.04 (.718)	-.01 (.962)	-.01 (.27)

Significant zero-order (*r*) and semi-partial (*sr*) correlations are highlighted in bold font (α = .05). *N* = 95. *Sr* represents the correlation between the habitual use of benign / malicious humor and the usage of a specific reappraisal strategy while generating humorous reappraisals, adjusted for the individual's productivity with regard to all (other) reappraisal strategies (i.e., the predominant implementation of a certain strategy). Parameter estimates (unstandardised regression coefficients B) are given along with their standard errors (SE).

### Reappraisal strategies implemented in the generation of humorous reappraisals of threatening situations and DSM-5 personality trait domains

A smaller share of reappraisals of the "positive reinterpretation" sub-type referring to finding positive aspects was generated by individuals scoring higher on Antagonism (*sr* = -.22, *p* = .034) and Negative Affectivity (*sr* = -.29, *p* = .004). Negative Affectivity was at the same time positively related to the generation of worst-case comparisons, which represents another subtype of the "positive reinterpretation" category (*sr* = .20, *p* = .045). Individuals higher on Detachment, too, produced more worst-case comparisons compared to the other reappraisal sub-types in the analysis (*sr* = .34, *p* = .001). A greater share of de-emphasizing reappraisals that entail handing over responsibility was related to higher scores on the personality trait domain of Psychoticism (*sr* = .20, *p* = .047). See Table 2 for a summary of the results. Again, the pattern of zero-order and semi-partial correlations indicates that the observed associations are primarily attributed to the propensity to generate relatively more reappraisals of a certain type compared to other types, rather than to the individuals' more general inventiveness in generating humorous cognitive reappraisals.

**Table 2:** Correlations between reappraisal strategies implemented in the generation of humorous reappraisals of threatening events and self-reported depressive experiences.

		Depression (CES-D)		
**Reappraisal strategies**		*r (p)*	*sr (p)*	*B (SE)*
**Positive re-interpretation**	General positive aspects	**-.23 (.023)**	**-.32 (.002)**	-4.37 (1.34)
	Worst-case comparison	**.21 (.039)**	**.31 (.002)**	4.73 (1.52)
	Disadvantage as advantage	.03 (.807)	.04 (.655)	.38 (.85)
**De-emphasising**	Alternative explanation	.04 (.727)	.04 (.724)	.14 (.39)
	Trivialising the problem	.04 (.669)	-.01 (.927)	-.75 (.81)
	Handing over responsibility	-.01 (.958)	-.02 (.813)	-.70 (2.95)

Significant zero-order (*r*) and semi-partial (*sr*) correlations are highlighted in bold font (α = .05). *N* = 95. *Sr* represents the correlation between depression and the usage of a specific reappraisal strategy while generating humorous reappraisals, adjusted for the individual's productivity with regard to all (other) reappraisal strategies (i.e., the predominant implementation of a certain strategy). Parameter estimates (unstandardised regression coefficients B) are given along with their standard errors (SE).

### Reappraisal strategies in the generation of humorous reappraisals of threatening events and self-reported depressive experiences

A greater share of positive reinterpretations featuring general positive aspects was related to greater psychological well-being in terms of less depressive experiences (*sr* = -.32, *p* = .002). By contrast, more depressive experiences were reported by participants having produced relatively more reinterpretations using worst-case comparisons (*sr* = .31, *p* = .002). See Table 3 for details.

**Table 3:** Correlations between reappraisal strategies implemented in the generation of humorous reappraisals of threatening events and DSM-5 personality trait domains.

		Negative affectivity			Detachment			Antagonism			Disinhibition			Psychoticism		
		*r (p)*	*sr (p)*	*B (SE)*	*r (p)*	*sr (p)*	*B (SE)*	*r (p)*	*sr (p)*	*B (SE)*	*r (p)*	*sr (p)*	*B (SE)*	*r (p)*	*sr (p)*	*B (SE)*
**Positive re-interpretation**	General positive aspects	**-.26 (.011)**	**-.29 (.004)**	-.20 (.07)	-.06 (.556)	-.18 (.078)	-.12 (.07)	**-.23 (.021)**	**-.22 (.034)**	-.15 (.07)	-.07 (.525)	-.04 (.688)	-.02 (.06)	-.05 (.632)	-.06 (.547)	-.04 (.07)
	Worst-case comparison	.18 (.098)	**.20 (.045)**	.15 (.07)	**.30 (.004)**	**.34 (.001)**	.25 (.08)	-.04 (.713)	.04 (.693)	.03 (.08)	-.01 (.916)	.01 (.916)	.01 (.07)	.12 (.247)	.14 (.169)	.10 (.07)
	Disadvantage as advantage	.03 (.798)	.06 (.515)	.03 (.04)	-.02 (.841)	-.02 (.845)	-.01 (.04)	-.13 (.206)	-.11 (.298)	-.05 (.04)	-.02 (.855)	-.01 (.892)	.01 (.04)	-.17 (.09)	-.14 (.186)	-.06 (.04)
**De-emphasising**	Alternative explanation	.09 (.369)	.04 (.662)	.01 (.02)	.01 (.993)	.01 (.975)	.00 (.02)	.05 (.616)	.03 (.803)	.01 (.02)	-.02 (.886)	-.06 (.548)	-.01 (.02)	.09 (.403)	.03 (.772)	.01 (.02)
	Trivialising the problem	.01 (.892)	-.02 (.871)	-.01 (.04)	-.04 (.674)	-.08 (.413)	-.03 (.04)	.04 (.725)	-.01 (.922)	.00 (.04)	.06 (.581)	.07 (.527)	.02 (.04)	.07 (.525)	.04 (.679)	.02 (.04)
	Handing over responsibility	**.21 (.044)**	.18 (.075)	.26 (.15)	-.01 (.969)	-.01 (.911)	-.02 (.14)	.12 (.244)	.08 (.441)	.12 (.15)	.18 (.088)	.19 (.079)	.23 (.13)	**.23 (.024)**	**.20 (.047**)	.29 (.14)

Significant zero-order (*r*) and semi-partial (*sr*) correlations are highlighted in bold font (α = .05). N = 95. *Sr* represents the correlation between the respective DSM-5 personality trait domain and the usage of a specific reappraisal strategy while generating humorous reappraisals, adjusted for the individual's productivity with regard to all (other) reappraisal strategies (i.e., the predominant implementation of a certain strategy). Parameter estimates (unstandardised regression coefficients B) are given along with their standard errors (SE).

### Supplementary analyses

Antagonism (*r* = .39, *p* < .001) but not Negative affectivity (*r* = .04, *p* = .671) was correlated with greater use of mean-spirited humour. Neither of these two personality trait domains was correlated with the use of benign humour (*r* = -.12, *p* = .246; *r* = -.15, *p* = .158). Greater use of malicious humour was also associated with higher scores on Detachment (*r* = .20, *p* = .052) and Psychoticism (*r* = .26, *p* = .011). For the intercorrelations between the habitual use of benign and malicious humour and the DSM-5 personality trait domains see Table 4.

**Table 4:** Intercorrelations between typical use of benign and malicious humour and DSM-5 personality trait domains.

	Benign humour	Malicious humour	Negative Affectivity	Detachment	Antagonism	Disinhibition	Psychoticism
**Malicious humour**	.10	-					
**Negative Affectivity**	-.15	.05	-				
**Detachment**	-.16	**.20**	.38	-			
**Antagonism**	-.12	**.39**	.03	.20	-		
**Disinhibition**	-.12	.10	.43	.37	.27	-	
**Psychoticism**	-.02	**.26**	.20	.37	.43	.47	-

Significant zero-order (*r*) correlations are highlighted in bold font (α = .05). N = 95.

Relative use of positive reinterpretations featuring positive aspects was not correlated with the total rate of reappraisals classified as having a humorous structure (*r* = .10, *p* = .337; *sr* = -.02, *p* = .853). However, participants with a higher rate of humorous ideas produced a greater share of worst-case comparisons (*r* = .26, *p* = .012; *sr* = .25, *p* = .013).

Corroborating the validity of the CSM scales, in participants with greater self-reported use of malicious humour as a trait, a higher percentage of reappraisals implying disparagement humour was observed in the behavioural test (*r* = .23, *p* = .027), with non-significant correlations for incongruity-resolution and nonsense humour (*r* = .09, *p* = .368; *r* = -.11, *p* = .309). In participants with greater use of benign humour as assessed by the CSM, the percentage of humorous reappraisals classified as incongruity-resolution humour was slightly higher, but not statistically significant (*r* = .18, *p* = .082; nonsense humour: *r* = .03, *p* = .808; disparagement humour: *r* = -.12, *p* = .256).

## Discussion

The findings of the present study propose links of one's habitual use of benign and malicious humour and broader personality traits with the implementation of specific strategies in the generation of humorous cognitive reappraisals. The use of sub-strategies in the domains of positive reinterpretation and de-emphasising was assessed in a behavioural test in which participants were required to generate a series of humorous reappraisals of threatening events. While no robust relationships emerged for reappraisal strategies based on de-emphasising, individual strategies within the positive reinterpretation category showed a specific and contrasting pattern of associations with the examined traits. Previous studies have established links between good-natured humour and more effective emotion regulation [8] as well as scrutinized personality traits relevant to humour production and humour styles [34,35]. In addition to that evidence, the present study offers novel insights into individuals' implementation of humorous cognitive reappraisal when faced with stressful events by linking them with various traits considered eminently relevant in both, humour research and research on emotion regulation.

Individuals with greater habitual use of benign and individuals with lesser habitual use of malicious humour specifically produced a greater share of positive reinterpretations referring to finding positive aspects. Greater use of this specific reappraisal strategy, in turn, was associated with less depressive experiences in everyday life. Here, it is important to note that while the research background of this study suggests cognitive reappraisal implementation as the cause and depressive experiences as the effect [54,55], this relationship may be bidirectional, with other studies reporting that depressive episodes may hamper effective emotion regulation [56]. The total amount of successfully produced humour responses in the task did not correlate with the generation of reinterpretations featuring positive aspects. This indicates that the links to this reappraisal strategy can be attributed to individuals' typical use of different kinds of humour but not to their general ability to produce humour in the context of cognitive reappraisal of stressful situations. This finding is in line with previous evidence indicating that, on its own, an enhanced sense of humour does not automatically translate to greater well-being [57–63].

An open question is why precisely the implementation of general positive aspects in cognitive reappraisal is related to the assessed humour traits. One may argue that generating humorous reappraisals primarily based on positive situational characteristics requires a greater amount of positive refocusing than mentally concocting a considerably worse situation (worst-case comparison) or imagining a personal gain from a negative situation (interpreting a disadvantage as an advantage). The fundamental positive and negative thinking biases associated with the habitual use of benign and malicious humour, respectively, may facilitate / interfere with overcoming the more difficult positive refocusing challenge [64]. The findings suggest that a more favourable pattern of reappraisal strategies may contribute to the association between the habitual use of benign forms of humour and greater life satisfaction, which is mediated by the establishment of robust positive affectivity [3,65].

In terms of more general personality traits, lesser generation of reappraisals featuring general positive aspects in the situation was associated with higher expressions of antagonism and negative affectivity. Antagonism represents the maladaptive extension of low levels of the classic "Big Five" trait agreeableness [37,38], while negative affectivity represents the maladaptive extension of neuroticism [37,38]. Important to note, the correlational pattern between the habitual use of benign and malicious humour and the personality trait domains indicated that the associations between this specific reappraisal strategy and the typical use of humour did not simply mirror the effects of the broader personality traits. Only antagonism, but not negative affectivity correlated with the use of malicious humour. Use of benign humour was not correlated with either of the DSM-5 personality trait domains.

Beyond antagonism, greater use of mean-spirited humour was additionally correlated with higher scores on detachment (representing the maladaptive extension of low extraversion) and psychoticism (largely representing the maladaptive extension of openness) [37,38]. This pattern of correlations can be integrated in extant literature reporting links between aggressive humour styles and related maladaptive personality traits such as antagonistic, callous, manipulative, narcissistic, avoidant personality as well as associated early maladaptive schemas [41, 66–69].

While the habitual use of benign and malicious humour was only linked to the share of reappraisals featuring positive aspects, the DSM-5 personality trait domains also exhibited associations with the generation of another subtype of the "positive reinterpretation" category, namely worst-case comparisons. A greater share of worst-case comparisons was linked to higher scores on negative affectivity and detachment. At the same time, the generation of more reinterpretations implicating worst-case comparisons was related to more depressive experiences in daily life.

Interestingly, while the seemingly unfavourable reappraisal strategy of worst-case comparisons was not linked to individuals’ habitual use of different kinds of humour, it was associated with a higher total rate of reappraisals classified as having a humorous structure. This pattern differs from the associations found for the favourable reappraisal strategy of reinterpretations featuring positive aspects, which was linked to greater habitual use of benign humour and lesser use of malicious humour, but did not show a relationship to the total ability of producing humour in the humorous reappraisal task. Together, these findings corroborate the notion that the overall ability to produce humour in the context of cognitive reappraisal alone is not linked to a favourable pattern of reappraisal strategies, and may even be related to adverse outcomes if not clearly manifested in benign forms of humour. The latter is in accordance with previous research suggesting that the habitual use of malicious humour, including self-disparaging forms, may be detrimental to psychological well-being [66,70–75]. Studies in professional comedians suggested that lower levels of concern and the denial of problems that may be associated with a highly humorous attitude might pose a risk to people with extraordinary ability to produce humour [61,76,77]. Important in this context, evidence indicated that in opposition to other forms of (humour) coping, humorous cognitive reappraisal of stressful situations does not mean to simply distract oneself from the adversity and deny it, thus still allowing adaptive dealings with the problem [7]. This may be particularly true for certain reappraisal strategies such as the positive reinterpretation subtype referring to finding positive aspects, and less so for worst-case comparisons.

Together, the analyses yielded a reasonable and coherent picture, which can be well integrated in the extant literature. Nevertheless, all statistical relationships in this explorative study should of course be treated with appropriate restraint until complemental research and replication allows for drawing firm conclusions. Moreover, due to the correlational/cross-sectional design of this study, causality and direction of influences cannot be directly inferred. Finally, this study used a sample of young, rather well educated adults recruited in college and university settings. Thus, replication in larger, more representative samples composed of different age groups and educational backgrounds is needed, before far-reaching conclusions can be drawn.

In summary, the study contributes to bridging research on humour and emotion regulation, by indicating that some characteristics that advance the use of benign humour also benefit adaptive emotion regulation. The reverse seems to be true for malicious, or "dark" humour. Along these lines, further research is warranted to determine which characteristics and possibly, which neurocognitive functions may underline these associations. Last but not least, the present study introduced a promising and powerful method for the profound investigation of humorous cognitive reappraisal of adverse circumstances, which may prove useful in future related research.

## Supporting information

S1 Appendix Reappraisal sub-strategies scored in this study. (DOCX)

S1 Dataset (XLSX)
