# Supplementary material for: Humorous cognitive reappraisal: More benign humour and less "dark" humour is affiliated with more adaptive cognitive reappraisal strategies
Source: PLoS One. 2019 Jan 31;14(1):e0211618. doi: 10.1371/journal.pone.0211618 (PMC6355006; doi:10.1371/journal.pone.0211618)
Supplement: S1 Appendix — (DOCX) [file pone.0211618.s001.docx]

**Appendix: Reappraisal sub-strategies scored in this study**(Weber et al., 2014; de Assuncao et al., 2015)

1. **Positive re-interpretation:** finding general positive aspects

*Emphasizing general positive characteristics of a situation are not directly related to the threatening experience, e.g., appreciating the surroundings or the accomplishments of the day*

1. **Positive re-interpretation:** worst-case comparison

*Directly comparing the threatening event to another, even more threatening event and in the process, appraising the current event as less threatening, e.g., it could always be worse*

1. **Positive re-interpretation:** interpret disadvantage as advantage

*Finding an advantage in a disadvantageous situation, e.g., a good opportunity to practice something*

1. **De-emphasising:** finding alternative explanations

*Finding another, non-threatening explanation for the current event, e.g., attributing the unpleasant situation to a rather harmless cause*

1. **De-emphasising:** trivializing the problem

*Downplaying the probability of the threatening event, e.g., what are the odds that this is happening?*

1. **De-emphasising:** handing over responsibility

*Not feeling responsible for the threatening situation, handing over responsibility to others, e.g., somebody will likely come and remedy the situation*
